# Larvicidal Activity of Carbon Black against the Yellow Fever Mosquito *Aedes aegypti*

**DOI:** 10.3390/insects13030307

**Published:** 2022-03-20

**Authors:** Erick J. Martínez Rodríguez, Parker Evans, Megha Kalsi, Noah Rosenblatt, Morgan Stanley, Peter M. Piermarini

**Affiliations:** 1 Ohio Agricultural Research and Development Center, Department of Entomology, The Ohio State University, Wooster, OH 44691, USA; martinezrodriguez.2@buckeyemail.osu.edu; 2 Indiana Center for Regenerative Medicine & Engineering, Indiana University Health Comprehensive Wound Center, Department of Surgery, Indiana University School of Medicine, Indianapolis, IN 46202, USA; evanspa@iu.edu; 3 Lepidext Inc., Lexington, KY 40505, USA; kalsimegha@gmail.com; 4 Vaylenx, LLC, Washington, DC 20003, USA; nrose2213@gmail.com (N.R.); morganstanley@gmail.com (M.S.)

**Keywords:** nanoparticles, mosquito, larvicide, insecticide resistance, carbon black

## Abstract

**Simple Summary:**

Nanoparticles have previously shown potential to control mosquito vectors. The present study examined whether carbon black, an industrial source of carbon-based nanoparticles (CNPs), was toxic to larvae of the yellow fever mosquito (*Aedes aegypti).* We found that exposing the first developmental stages of mosquito larvae to a modified form of carbon black EMPEROR^®^ 1800 (E1800), caused concentration-dependent mortality within 48 h of exposure; however, the development of larvae exposed to sub-lethal concentrations of E1800 was not disrupted. Analyses of E1800 suspensions suggest this carbon black forms CNPs that coalesce into larger aggregations. Microscopic observations of dead larvae showed the presence of CNP aggregations in the digestive tract and on external structures associated with swimming, breathing, and food uptake. Our results suggest carbon black is a source of CNPs that may have potential use for treating sources of standing water that mosquitoes use as breeding sites.

**Abstract:**

The yellow fever mosquito *Aedes aegypti* is one of the deadliest animals on the planet because it transmits several medically important arboviruses, including Zika, chikungunya, dengue, and yellow fever. Carbon-based nanoparticles (CNPs) derived from natural sources have previously been shown to have toxic effects on mosquito larvae and offer a potential alternative to chemical insecticides such as pyrethroids, for which mosquitoes have evolved resistance. However, CNPs derived from industrial sources, such as carbon black, have not previously been evaluated as larvicides. Here, we evaluate the effects of a commercially-available carbon black, EMPEROR^®^ 1800 (E1800), on mortality and development of pyrethroid-susceptible (PS) and pyrethroid-resistant (PR) strains of *Ae. aegypti*. We found that E1800 exhibited concentration-dependent mortality against 1st instar larvae of both strains within the first 120 h after exposure, but after this period, surviving larvae did not show delays in their development to adults. Physical characterization of E1800 suspensions suggests that they form primary particles of ~30 nm in diameter that fuse into fundamental aggregates of ~170 nm in diameter. Notably, larvae treated with E1800 showed internal accumulation of E1800 in the gut and external accumulation on the respiratory siphon, anal papillae, and setae, suggesting a physical mode of toxic action. Taken together, our results suggest that E1800 has potential use as a larvicide with a novel mode of action for controlling PS and PR mosquitoes.

## 1. Introduction

The yellow fever mosquito *Aedes aegypti* (Linnaeus, 1762) is a vector of many arboviruses that debilitate human health worldwide, including chikungunya, dengue, yellow fever, and Zika. Each year, hundreds of millions of people are infected with these viruses, leading to millions of hospitalizations and tens of thousands of deaths [1,2,3,4,5,6]. In the absence of effective and widely available vaccines, limiting transmission of these viruses often relies on controlling the primary mosquito vector. Despite recent advances in biological and genetic control of mosquitoes, chemical control via adulticides (e.g., pyrethroids) and larvicides (e.g., pyriproxyfen) remains a commonly used approach to control mosquito populations, especially during emerging outbreaks of arboviruses [7]. However, the overuse of chemicals with limited modes of action has led to resistance in *Ae. aegypti* and other mosquito vectors [7,8]. Thus, the discovery of insecticides with novel modes of action are needed to improve the chemical control of mosquito vectors.

Recently, nanoparticles have shown potential to control mosquito vectors. Nanoparticles range in size between 1–100 nm and can consist of novel shapes, surface compositions, and solubility. The physical and chemical properties of nanoparticles can be tailored to interact with various cellular structures or deliver ‘cargo’ to elusive biological targets, making them potentially exciting materials for developing novel and safe tools for mosquito control [9,10,11]. Previous studies have shown that nanoparticles possess insecticidal activity against larval and pupal stages of mosquitoes [12,13,14,15,16]. Moreover, nanoparticles have been used as delivery systems for dsRNA and siRNA to silence the expression of genes [10,17,18,19]. Furthermore, studies have shown that various non-target organisms that live in the same aquatic environments as mosquito larvae are seldomly impacted by exposure of different types of nanoparticles [20,21,22,23,24,25,26].

Carbon-based nanoparticles (CNPs) have recently been evaluated as potential larvicidal agents for mosquito control [26]. CNPs are recognized for their unique properties including outstanding electrical conductivity, mechanical strength, structural stability, and high surface area [27,28] The process of synthesizing CNPs has improved throughout the years using a variety of techniques including carbon-arc vaporization, catalytic decomposition of organic vapors, or laser vaporization of organic and inorganic resources [29]. Plant-derived CNPs have been shown to be effective larvicides against mosquitoes [26,30,31], but there is limited research available on the efficacy of CNPs from industrial sources, such as carbon black, as potential larvicides.

Carbon black is a nanostructured carbon material composed of elemental carbon and is frequently used as a pigment, rubber reinforcer, ultraviolet protectant, and conductive agent in a variety of elastomers, plastics, and coatings [29,32,33]. Although there are environmental and human health concerns over carbon black [34,35,36,37], more than 10 million metric tons are produced annually using the furnace black method, making it a remarkably abundant source of a potential mosquito control agent. The furnace black method involves the thermal-oxidative decomposition of aromatic oils on coal tar, mineral oil, or natural gas and creates carbon black with primary particle sizes ranging from 10 to 80 nm. Primary particle size, aggregate size, and surface activity are the fundamental properties of carbon black that make this material efficient and versatile in its implementation on various uses [29]. However, to our knowledge, it has not previously been evaluated for insecticidal activity against mosquitoes.

The goal of this study was to test the larvicidal efficacy of a modified-form of carbon black against pyrethroid-susceptible (PS) and pyrethroid-resistant (PR) strains of the yellow fever mosquito *Ae. aegypti*. We evaluated the commercially available carbon black, EMPEROR^®^ 1800 (E1800; Cabot Corporation, Boston, MA, USA), which is widely used in formulations for water-based automobile coatings. We hypothesized that the ability of E1800 to fully disperse in water would disrupt larval mosquito survival and/or development.

## 2. Materials and Methods

### 2.1. Mosquito Rearing Conditions

*Ae. aegypti* mosquito larvae were reared following established protocols [38,39]. Eggs of the Liverpool and Puerto Rico strains of *Ae. aegypti* were obtained through the MR4 as part of the BEI Resources Repository, NIAID, NIH (LVP-IB12, MRA-735, deposited by M.Q. Benedict; PR, NR-48830, deposited by G.G. Clark & J.J. Becnel). Larvae of the Puerto Rico strain are over 100-fold resistant to the pyrethroid cypermethrin compared to the Liverpool strain [39]. From herein, we refer to the Liverpool and Puerto Rico strains as the pyrethroid-susceptible (PS) and pyrethroid-resistant (PR) strains, respectively. To maintain the resistance trait in the PR strain, 3rd instar larvae were treated every third generation with 0.1 mg/mL cypermethrin (Acros Organics, Geel, Belgium) for 10–15 min until ~50% of larvae were immobilized. Larvae of all strains were fed ground fish food flakes (Tetramin, Melle, Germany). Adult mosquitoes of both strains were fed 10% sucrose solution ad libitum. Additional eggs were produced as needed by feeding adult females defibrinated rabbit blood (Hemostat Laboratories, Dixon, CA, USA) with a membrane feeder (Hemotek, Blackburn, UK). All mosquitoes were maintained in environmentally controlled rearing chambers (28 °C, 80% relative humidity, 12 h:12 h light:dark cycle).

### 2.2. Preparation of E1800

Unless indicated otherwise, fresh 10 mg/mL stock suspensions of E1800 were prepared on the day of an experiment. In brief, 0.1 g of Modified Carbon Black EMPEROR^®^ 1800 (CABOT, Pampa, TX, USA) was added to 10 mL of distilled water (dH_2_O) in a 20 mL scintillation vial (ThermoFisher Scientific™, Waltham, MA, USA) and vortexed vigorously for 20 s. The E1800 suspensions used for the experiments to evaluate larval mortality and development were prepared by diluting the 10 mg/mL stock with dH_2_O.

### 2.3. Acute Toxicity Assay: 1st Instar Larvae

To determine the acute (within 48 h) toxicity of E1800 against 1st instar larvae, we used an established assay [39]. Briefly, five 1st instar *Ae. aegypti* larvae of both PS and PR strains were placed in wells of a 24-well Falcon^®^ Multiwell plate (Becton Dickinson Labware, Franklin Lakes, NJ, USA) containing 995 μL of a E1800 suspension and 5 μL of food solution (13 mg/mL of finely ground fish food flakes in dH_2_O; Tetramin, Blacksburg, VA, USA). The final concentrations of E1800 used to generate a concentration-response curve (CRC) were 0.01, 0.1, 0.5, 1.0, 2.5, 5.0, or 10.0 mg/mL. Control wells received 995 μL of dH_2_O and 5 μL of food solution. All plates were held under standard rearing conditions for 48 h before larval mortality was evaluated. Larvae were considered dead if they did not move after gently touching their abdomen with a fine needle or pipette tip. All mortality values were corrected for control mortality using Abbott’s formula [40]. On average, control mortality in the PS and PR strains was 1.6% and 3%, respectively.

### 2.4. Acute Toxicity Assay: 3rd Instar Larvae and Pupae

Groups of three 3rd instar larvae or pupae were placed in each well of a 24-well Falcon^®^ Multiwell plate containing 990 μL of E1800 solution (0.1, 1, or 5 mg/mL) and 10 μL of food solution. Control wells received 995 μL of dH_2_O and 5 μL of food solution. Acute mortality of these life stages was assessed 48 h after E1800 exposure by considering them dead if they did not move after gently touching their abdomen with a fine needle or pipette tip. All mortality values were corrected for control mortality using Abbott´s formula [40]. In these experiments we observed no control mortality in the PS or PR strains.

### 2.5. Stability of Acute Toxicity of E1800 against 1st Instar Larvae

To evaluate whether the acute toxicity of E1800 suspensions in water changed over time, we exposed 1st instar *Ae. aegypti* larvae to different concentrations of the same E1800 stock that had been prepared 0 (fresh), 2, 4, 8, or 12 weeks prior to an experiment. Acute toxicity was determined at 48 h as described in ‘Acute toxicity assay: 1st instar larvae’ above. The final E1800 concentrations used for these experiments were 0.1, 1.0, or 5.0 mg/mL. Control wells received 995 μL of dH_2_O and 5 μL of food solution. All mortality values were corrected for control mortality using Abbott´s formula [40]. In these experiments we observed no control mortality in the PS or PR strains.

### 2.6. Chronic Toxicity and Development Assay: 1st Instar Larvae

To assess the toxic effects of E1800 on larvae beyond 48 h and determine potential effects on development into adults, we monitored survival of mosquito larvae and their emergence into adults for 10 days. Briefly, thirty 1st instar *Ae. aegypti* larvae of the PS or PR strain were placed in 150 mm × 25 mm Falcon^®^ petri dishes containing 100 mL of E1800 at 0.1 or 0.25 mg/mL. Control dishes received 100 mL of dH_2_O. Each dish was provided with 13 mg of pulverized fish food daily for the first seven days. After seven days, each dish received 20 mg of pulverized fish food daily to accommodate the increase of larval body size. Plates were held under standard rearing conditions. Larvae were examined daily for 14 days through an Olympus SZ6145TR Stereo Microscope (B&B Microscopes Limited, Pittsburg, PA, USA) equipped with a GO3 Digital Color Camera (Takakuramachi, Hachioji-shi, Tokyo). Larvae were considered dead if they did not move after gently touching their abdomen with a fine needle or pipette tip. Once pupae appeared in the dishes, the standard lids of the dishes were replaced with ones containing a funnel and a small receptacle with 10% sucrose-soaked cotton balls to collect any adults that emerged. Images of larvae were captured with the GO3 Digital Color Camera and edited using Olympus CellSens Entry 2.2 software (Olympus Corporation^®^, 2009–2018). All mortality values were corrected for control mortality using Abbott´s formula [40]. On average, control mortality in the PS and PR strains was 5% and 4%, respectively.

### 2.7. Physical Characterization of E1800 Suspensions

To measure the size of the nanoparticles derived from E1800, we used scanning electron microscopy (SEM) and the solvent casting method. In brief, E1800 was suspended in 200 proof ethanol at 0.5 mg/mL, spin coated onto an aluminum 12 mm slotted head specimen mount (TedPella Inc.,16111, Redding, CA, USA) and dried under nitrogen for 30 min. Adhesive carbon paper tabs measuring 12mm (TedPella Inc., Redding, CA, USA) were placed onto separate specimen mounts and a 50 nm layer of gold was deposited using a Quorum EMS 150R ES sputter coater (Quorum, Laughton, East Sussex, UK). Following sputter coating, the solvent-casted E1800 was adhered to the gold-sputtered adhesive specimen mounts by pressing the gold-sputtered specimen mounts to the solvent-casted specimen mounts and uniformly lifting the particles off the aluminum surface. All specimen mounts were cleaned with 100% isopropyl alcohol before use. The porosity of the carbon paper tabs led to some cracking of the gold layer especially where samples were exposed to the electron beam for longer durations. Specimens were visualized on a FEI Helios Nanolab 600 Dual Beam Focused Ion Beam/Scanning Electron Microscope (Field Electron and Ion Company, Hillsboro, OR, USA) in The Ohio State University Center for Electron Microscopy and Analysis (College of Engineering, Columbus, OH, USA).

In addition to SEM, we used a dynamic light scattering (DLS) approach to measure E1800 particle size [41,42]. In brief, E1800 was freshly prepared at a concentration of 1 mg/mL in 18.2 MΩ water. This stock suspension was diluted 1:100 with 18.2 MΩ water (MilliPore Direct 16, MilliporeSigma, Burlington, MA, USA) and placed in a borosilicate glass tube (12 mm outer diameter × 75 mm) (ThermoFisher Scientific™, Waltham, MA, USA) with a light path of 10 mm. Light scattering measurements were performed on a BI-200SM Goniometer (Brookhaven Instruments, Holtsville, NY, USA) at an angle of 90 degrees and a laser wavelength of 653 nm. Data were resolved on a PC and exported to MATLAB (The MathWorks, Inc., Natick, MA, USA) for plotting and analysis. Following DLS analysis, the same sample was transferred to a quartz cuvette (Brookhaven Instruments, Holtsville, NY, USA) for zeta potential determination on a BIC Zeta/Stream Potential Measurement System (Brookhaven Instruments, Holtsville, NY, USA). The electrode cap was secured, an electric field of 10.27 V/cm was applied, and 255,000 counts per second were taken for ~2 min until the zeta potential value stabilized to within 0.1% of the previous 100,000 readings. The mean zeta potential from five runs is reported.

### 2.8. Statistical Analysis

Data analysis and plotting were performed using GraphPad Prism (version 6.07) software (GraphPad, San Diego, CA, USA) and MATLAB (The MathWorks, Inc., Natick, MA, USA). To determine the median lethal concentration (LC_50_) or median lethal time (LT_50_), percent mortalities were first plotted against log transformations of concentrations or times, respectively. Then, the data were fitted with non-linear regressions using the ‘log(agonist) vs. normalized response’ function to calculate LC_50_ or LT_50_. Statistical comparisons of the LC_50_ or LT_50_ values were performed through F-tests. In experiments testing the stability of E1800 toxicity against larvae over time, percent mortalities were compared using a one-way analysis of variances (ANOVA) and Tukey´s multiple comparison test.

## 3. Results

### 3.1. E1800 Kills 1st Instar Larvae of PS and PR Strains within 48 h

**Figure 1:**
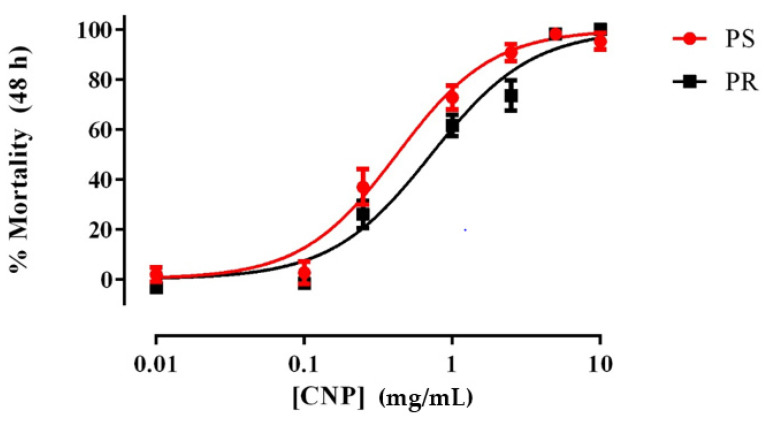
Concentration-response curves for acute toxicity (48 h) of E1800 against 1st instar Ae. aegypti of representative pyrethroid-susceptible (PS, red) and pyrethroid-resistant (PR, black) strains. Values plotted are means ± standard errors of the mean based on 12 replicates of 5 larvae per concentration (Control average PS: 2.5%; PR: 3.87%). The median lethal concentration (LC_50_) value of the PR strain (0.72 mg/mL) was significantly greater (F-test, *p* < 0.001) than that of the PS strain (0.42 mg/mL) as determined using a nonlinear regression fit and a F-Test (*p* < 0.001). Mortality values were corrected using Abbott´s formula [40].

As shown in Figure 1, freshly prepared suspensions of E1800 caused concentration-dependent mortality in 1st instar larvae of both PS and PR strains of *Ae. aegypti*. The LC_50_ value of the PR strain (0.72 mg/mL; 95% confidence interval [CI] = 0.60–0.86 mg/mL) was significantly greater (F-test, *p* < 0.001) than that of the PS strain (0.42 mg/mL; 95% CI = 0.33–0.53 mg/mL), suggesting a minor degree of resistance (~1.7 fold) of the PR strain to E1800 in this assay. We also assessed the acute toxicity of a few concentrations of E1800 (0.1, 1.0, or 5.0 mg/mL) against 3rd instar larvae and pupae, but none of these treatments resulted in mortality (Table S1). Thus, the larvicidal activity of E1800 does not manifest unless treatment begins when larvae are 1st instars.

### 3.2. Stability of the Larvicidal Activity of E1800 Suspensions

**Figure 2:**
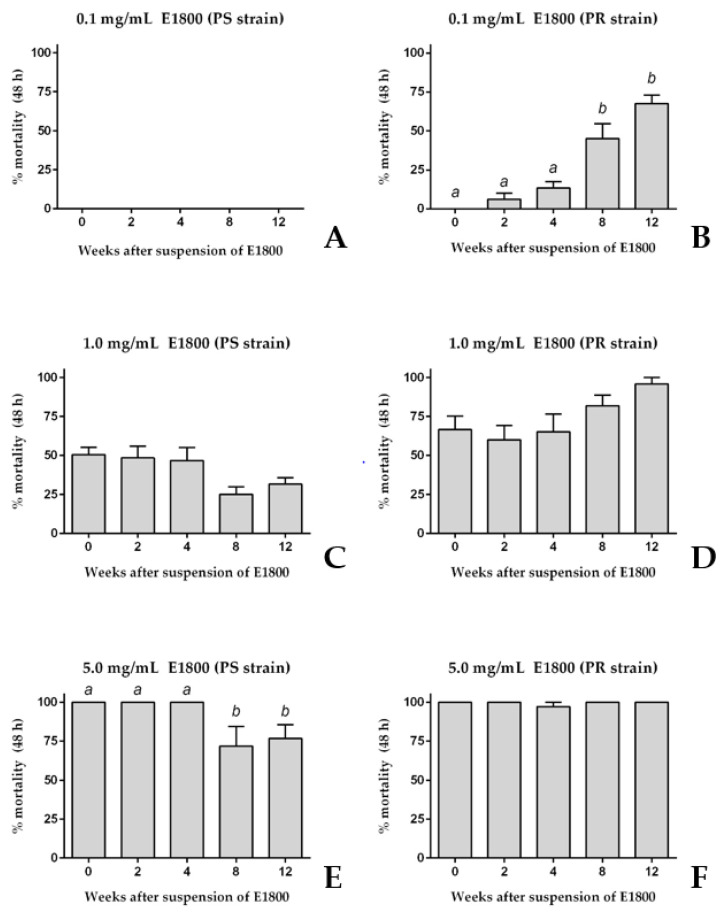
Acute toxicity (48 h) of E1800 suspensions against 1st instar *Ae. aegypti* of pyrethroid-susceptible (PS, panels (**A**,**C**,**E**)) and pyrethroid-resistant (PR, panels (**B**,**D**,**F**)) strains at several time points after being freshly suspended (week 0). Three different concentrations were tested: 0.1 mg/mL (**A**,**B**); 1.0 mg/mL (**C**,**D**); and 5.0 mL/mL (**E**,**F**). Values are means + standard errors of the mean based on six replicates of five larvae per concentration. Lower-case letters indicate statistical categorization of the means as determined using a one-way ANOVA and Tukey´s multiple comparison test (*p* < 0.001).

To determine if the acute toxicity of E1800 against 1st instar larvae was stable for several weeks after its suspension in water, we compared the toxic efficacy of E1800 suspensions (0.1, 1.0, or 5.0 mg/mL) at several time points after preparation over a 12-week period. Similar to the results of Figure 1, a freshly prepared suspension (week 0) of 0.1 mg/mL E1800 did not kill 1st instar larvae of the PS strain; this suspension remained non-toxic for the next 12 weeks (Figure 2A). Likewise, the freshly prepared 0.1 mg/mL E1800 suspension was non-toxic to PR larvae and remained non-toxic for the next 4 weeks after suspension (Figure 2B). However, at weeks 8 and 12 after suspension the acute toxicity of this suspension remarkably increased against PR larvae to ~45% and ~70% mortality, respectively. 

Higher concentrations of E1800 showed less remarkable changes in toxicity over time. Similar to the results of Figure 1, a freshly prepared suspension of 1.0 mg/mL E1800 induced ~50% mortality in both the PS and PR strains (Figure 2C,D); the toxicity of this suspension did not significantly change over the remaining 12-week period (Figure 2C,D). Furthermore, similar to the results of Figure 1, a freshly prepared suspension of 5.0 mg/mL E1800 induced ~100% mortality in both the PS and PR strains (Figure 2E,F); the toxicity of this suspension against the PS strain was similar within 4 weeks after suspension, but significantly decreased to ~75% mortality at weeks 8 and 12 after suspension (Figure 2E). On the other hand, the toxicity of this suspension did not significantly change in the PR strain in the remaining 12-week period (Figure 2F).

Taken together, the results of Figure 1 and Figure 2 are consistent in that freshly prepared suspensions of E1800 induce similar degrees of acute toxicity against 1st instar larvae of PS and PR strains of *Ae. aegypti*. However, as the E1800 suspension ages, its toxicity against the PR strain appears to increase or remain the same, whereas its toxicity against the PS strain appears to decrease or remain the same (Figure 2).

### 3.3. E1800 Is Toxic to Larvae within 120 h of Exposure and Does Not Alter Development Thereafter

**Figure 3:**
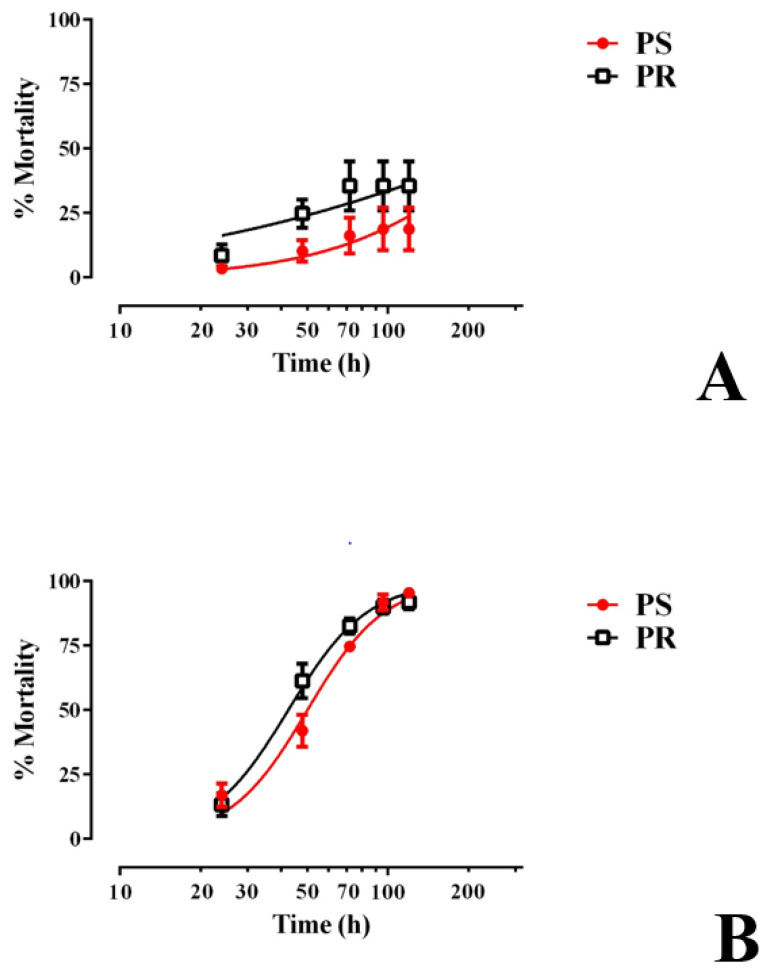
Chronic larval toxicity of E1800 against representative pyrethroid-susceptible (PS, red) and pyrethroid-resistant (PR, black) strains of *Ae. aegypti*. E1800 was tested at concentrations of (**A**) 0.1 mg/mL or (**B**) 0.25 mg/mL. The ‘x axis’ in both panels is log transformed and represents the time in which larvae were exposed to E1800 up to 120 h; points after this time remained consistent until the experiment was finalized (14 days = 264 h). Values plotted are means ± standards errors of the mean based on four replicates of 30 larvae for PS or six replicates of 30 larvae for PR. At 0.25 mg/mL E1800, the median lethal time (LT_50_) of the PR strain (42.5 h, 38.7–46.6 h 95% CI) was significantly lower (F test, *p* < 0.05) than that of the PS strain (50.1 h, 45.9–54.6 h 95% CI).

We next determined whether E1800 was toxic to larvae beyond the first 48 h of treatment by rearing groups of 1st instar larvae (30 per treatment) in the presence of E1800 (0.1 or 0.25 mg/mL) and monitoring their survival and development to adults. As shown in Figure 3A, larval mortality induced by 0.1 mg/mL E1800 was limited to 18–35% within the first 120 h of exposure for both strains and did not increase thereafter. All surviving larvae continued development to adults in a similar fashion as controls (Figure S1).

In the presence of 0.25 mg/mL E1800, larval mortality progressively increased every 24 h for the first 120 h in both the PS and PR strains, reaching 90–95% mortality (Figure 3B). The LT_50_ of the PR strain (42.5 h, 38.7–46.6 h 95% CI) was significantly lower (F test, *p* < 0.05) than that of the PS strain (50.1 h, 45.9–54.6 h 95% CI), indicating that, in this assay, larvae of the PR strain are slightly more susceptible to E1800 than those of the PS strain. The larvae that survived this treatment continued development to adults in a similar fashion as controls (Figure S1).

**Figure 4:**
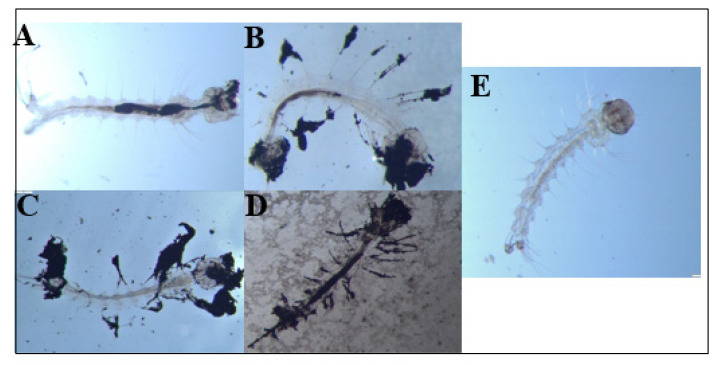
Dead *Ae. aegypti* larvae exposed to (**A**) 0.1 mg/mL or (**B**–**D**) 0.25 mg/mL E1800. For comparison, a control larva is shown in panel (**E**). Accumulation of black material is observed internally (midgut, head/mouthparts) and externally (setae, anal papillae) in the larval body. Larvae were observed under an Olympus SZ6145TR Stereo Microscope at a magnification of 25× and images were captured with a GO3 Digital Color Camera.

During these chronic exposure experiments, we also periodically removed dead larvae from the E1800 treatments and observed them under a dissecting microscope to look for potential causes of death. We documented accumulations of black material internally in the larval alimentary canal as well as externally attached to the head/mouthparts, setae, respiratory siphon, and anal papillae (Figure 4A–D). In larvae from controls, no accumulations of black material were observed (Figure 4E).

### 3.4. E1800 Suspensions Contain Nanoparticles That Form Fundamental Aggregates

**Figure 5:**
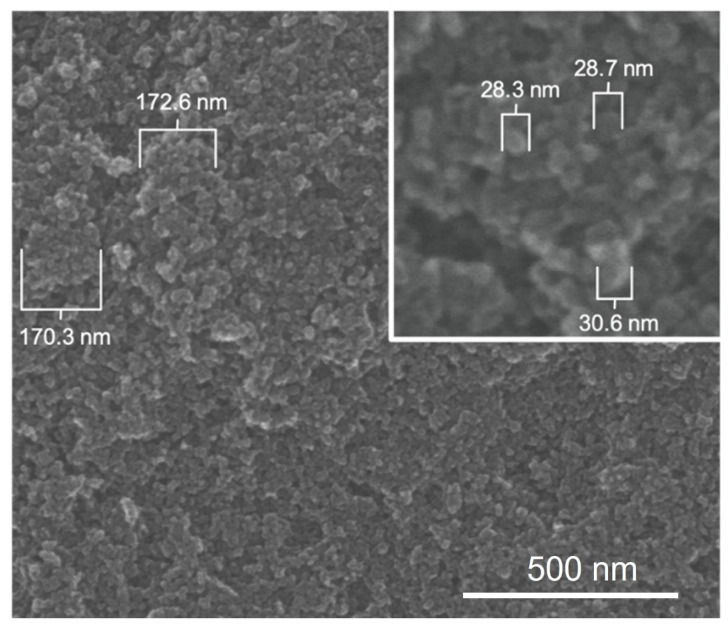
Scanning electron micrographs of E1800 (solvent casting method) showing primary carbon nanoparticles approximately 29 nm in diameter (inset) and aggregates of approximately 170 nm formed by primary particles.

**Figure 6:**
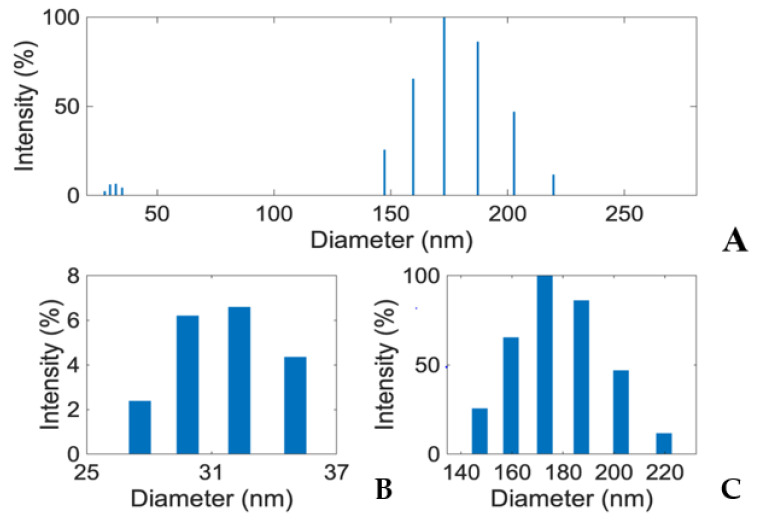
(**A**) Dynamic Light Scattering (DLS) analysis showing two distinct peaks (~32 nm and ~170 nm) in E1800 suspension (1 mg/mL). Mean particle diameter was 169.8 nm with a relative variance of 0.049 and a root mean squared error of 3.2 × 10^−2^. Panels (**B**,**C**) respectively show zoomed in views of the particle size (25–37 nm) and aggregate size (140–220 nm) distributions from the DLS analysis in panel (**A**). Note that larger particles/aggregates scatter light more effectively than smaller particles. Thus, the exact quantitative proportions of the various particles and aggregates cannot be determined from DLS intensity.

To generate insights into how E1800 may interact with mosquito larvae and induce mortality, we characterized physical properties of E1800 suspensions. Scanning electron micrographs (using the solvent casting method) suggested that E1800 forms individual nanoparticles of ~30 nm diameter that fuse into fundamental aggregates of ~170 nm (Figure 5). A DLS analysis of E1800 suspended in water (1 mg/mL) revealed a bimodal distribution of nanoparticle sizes with peaks at approximately 29 nm and 170 nm (Figure 6). These peaks are in good agreement with the respective sizes of the particles and aggregates determined in the SEM analysis. We also measured the zeta potential of the E1800 suspension, which was −8.25 mV ± 0.74 mV (mean ± standard error of the mean). This potential suggests that the aggregation of particles is due to attraction of the hydrophobic carbon nanoparticles coupled with stabilization by electrostatic repulsion [43,44]. In the absence of additional forces, the zeta potential suggests that nanoparticle aggregates are likely limited to sizes of less than 220 nm due to the electrostatic repulsion of the particulate surface charge in water, which is consistent with our observations in the SEM and DLS analyses (Figure 5 and Figure 6).

## 4. Discussion and Conclusions

The present study is the first to demonstrate that carbon black is toxic to mosquito larvae. Our results suggest that suspensions of a modified form of carbon black (E1800) in water form nanoparticles and primary aggregates that kill 1st instar larvae of PS and PR strains of *Ae. aegypti* in a concentration-dependent manner. However, the toxicity of E1800 was only effective when applied against 1st instar larvae and E1800 did not have detectable developmental effects on larvae. Our results are consistent with those of previous studies that suggest a variety of nanoparticles have potential use as larvicides [12,16,18,26,30,31,45,46,47,48]. However, our results contrast with previous studies that found nanoparticles can disrupt the development of mosquito larvae. For example, Saxena et al. [31] found that water-soluble carbon nanoparticles (wsCNP) synthesized from burnt wood wool ashes not only had potent concentration-dependent toxicity (2.0 mg L^−1^ produced ~30–35% mortality) against larval *Anopheles*, *Culex*, and *Aedes*, but also impaired the growth and development of larvae that survived wsCNP treatment at a concentration of 3.0 mg L^−1^. Moreover, Barik et al. [49] found that silica-based nanoparticles (siNPs), which share similar physical properties as carbon-based nanoparticles, were weakly toxic against 3rd instar mosquito larvae of *Ae. aegypti* compared to silver-based nanoparticles (AgNPs), but the siNPs elicited pupicidal activity and inhibited development [49]. Thus, the mode of toxicity of E1800 against mosquito larvae appears unique compared to other types of nanoparticles.

Although the specific mode of toxic action of E1800 on mosquito larvae is unknown, our results suggest that a physical component is likely involved. Notably, as observed in Figure 4, we documented larvae with E1800 particles in their alimentary canal, which indicates that the particles are ingested. Based on the bimodal distribution of sizes obtained from the SEM and DLS analyses (Figure 5 and Figure 6), we speculate that smaller particles are ingested by larvae during feeding, and compressive forces in the larval gut facilitate the formation of larger aggregates inside the alimentary canal where they may physically disrupt the passage and digestion of food and/or nutrient absorption by the midgut epithelium. Moreover, we observed larvae with E1800 aggregates attached to their head, abdominal setae, anal papillae, and respiratory siphon. The accumulation of aggregates on these vital structures could potentially impede feeding, locomotion, ionic regulation, and/or respiration. Previous histological studies of mosquito larvae treated with various nanoparticles have observed tissue damage to anal papillae, caeca, nerve cords, and muscles [50,51,52]. Whether E1800 elicits similar tissue damage remains to be determined.

Our experiments demonstrated that 1st instar larvae are most susceptible to E1800; the majority died within 48–72 h of treatment (Figure 3). After this time point, the mortality and effects of E1800 on remaining development are nominal, suggesting that once larvae reach a certain developmental stage or body size then they are no longer susceptible to the E1800 particles. Consistent with this notion, direct treatment of 3rd instar larvae or pupae with E1800 is without toxic effect. These findings suggest that E1800 would have the most potential use as a preventative treatment of larval breeding grounds to be applied prior to oviposition to ensure 1st instar larvae are exposed immediately upon hatching from eggs. Although not tested in the present study, it would be interesting for future studies to determine whether E1800 affects hatching efficiency or has any direct toxicity to mosquito eggs deposited into or near water with E1800.

One of the more intriguing and enigmatic findings of the present study was the apparent selective increase of toxic potency of E1800 suspensions over time to 1st instar larvae of the PR strain. As shown in Figure 2, freshly prepared suspensions of E1800 (0.1 mg/mL) exhibited similar toxic efficacy against PS and PR larvae, but after several weeks, the toxicity of E1800 remarkably increased against the PR strain. Conversely, the 5.0 mg/mL E1800 suspension showed a slight decrease of toxicity to the PS strain after several weeks, but remained highly effective against the PR strain, again suggesting a greater toxicity against the PR strain after several weeks in suspension. We are unable to directly explain this phenomenon, but presume it involves a change in the physical composition of the E1800 (e.g., particle size, aggregate size, zeta potential) over time when suspended in water. These results are consistent with those of a recent study that found time-dependent increases in cytotoxicity of carbon black against mammalian cells [53].

It is unclear why such potential changes would selectively increase toxicity to the PR over the PS strain, but it is widely appreciated that mutations contributing to resistance of insects to chemical insecticides (e.g., pyrethroids) are often associated with fitness costs in the absence of a selective pressure from the chemical [54]. In our case, the PR strain has been selected for resistance to a pyrethroid (cypermethrin) and not carbon black. Thus, the PR strain may carry a fitness cost that makes 1st instar larvae more susceptible to the presumed physical changes in the E1800 suspension as it ages. The specific changes to the E1800 suspension and fitness cost in the PR strain remain to be determined.

Despite our inability to explain the above increased toxicity of E1800 to the PR strain over time, the phenomenon carries potential applied relevance if an E1800-like product were to be used as a larvicide in the field. That is, our findings suggest that mosquito breeding sites could potentially be treated with E1800 several weeks in advance of oviposition and the water would remain toxic to 1st instar larvae. Moreover, the E1800-treated water would be most toxic to pyrethroid-resistant individuals, perhaps facilitating the management of pyrethroid resistance in mosquito populations. If the toxic properties of an E1800-like insecticide are specific to mosquitoes, which remains to be determined, then we could envision this application being especially useful for treating predictable mosquito breeding sites, such as bird baths near residences and storm water catch basins.

Even though nanoparticles appear to have low impact on non-target organisms, there are still many concerns regarding their use in terrestrial and aquatic environments due to the limited information available [26]. Carbon black is no exception. At the present time, we do not promote the use of E1800 or other carbon black-derived products as larvicides for mosquito control, given that other studies have raised strong environmental and human health concerns about carbon black [34,35,36]. Thus, any carbon-black-derived insecticide would require a thorough vetting for human and environmental safety. However, our study at least provides proof of concept that carbon black exhibits novel toxic properties against mosquito larvae with potential practical advantages (e.g., toxic activity for several weeks, resistance-mitigating activity) that warrant further investigation into the possibility of developing safe forms of carbon black for mosquito control.

## Data Availability

Data is contained within the article or supplementary material.

## Supplementary Materials

The following supporting information can be downloaded at: https://www.mdpi.com/article/10.3390/insects13030307/s1, Table S1. Mortality of 3rd instar larvae and pupae exposure to E1800 for 48 h. No mortality was observed in pyrethroid-susceptible (PS) or pyrethroid-resistant (PR) strains (*n* = six replicates containing three larvae and three pupae per treatment). Values are means ± standard errors of the mean. Figure S1. Adult emergence of *Ae. aegypti* pyrethroid-susceptible (PS) and pyre-throid-resistant (PR) strains after E1800 exposure at 0.1 mg/mL. The ‘x axis’ indicates the E1800 concentrations used against larvae of each strain. The ‘y axis’ represents the day at which 50% of adult emergence was attained. Values are means + standard errors of the mean. There were no significant differences observed between treatments and their respective controls (one-way ANOVA).
