# Supplementary material for: Larvicidal Activity of Carbon Black against the Yellow Fever Mosquito Aedes aegypti
Source: Insects. 2022 Mar 20;13(3):307. doi: 10.3390/insects13030307 (PMC8954748; doi:10.3390/insects13030307)
Supplement: Supplementary file 1 [file insects-13-00307-s001.zip › insects-1608891-supplementary.pdf]

Supplementary Materials:

Table S1. Mortality of 3rd instar larvae and pupae exposure to E1800 for 48 h. No mortality was observed in pyrethroid-susceptible (PS) or pyrethroid-resistant (PR) strains (n=6 replicates containing 3 larvae and 3 pupae per treatment). Values are means ± standard errors of the mean.

E1800 mg/ml	3rd Instar PR	Pupae PR	3rd Instar PR	Pupae PR
0.1	0% ± 0	0% ± 0	0% ± 0	0% ± 0
1	0% ± 0	0% ± 0	0% ± 0	0% ± 0
5	0% ± 0	0% ± 0	0% ± 0	0% ± 0

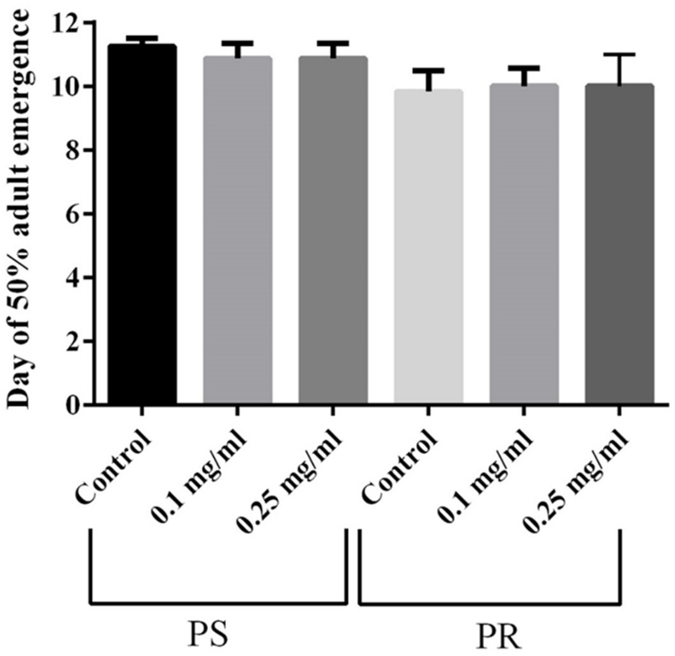

Figure S1. Adult emergence of *Ae. aegypti* pyrethroid-susceptible (PS) and pyrethroid-resistant (PR) strains after E1800 exposure at 0.1 mg/ml. The 'x axis' indicates the E1800 concentrations used against larvae of each strain . The 'y axis' represents the day at which 50% of adult emergence was attained. Values are means + standard errors of the mean . There were no significant differences observed between treatments and their respective controls (one-way ANOVA).
